# Feature Selection and Combination of Information in the Functional Brain Connectome for Discrimination of Mild Cognitive Impairment and Analyses of Altered Brain Patterns

**DOI:** 10.3389/fnagi.2020.00028

**Published:** 2020-02-19

**Authors:** Xiaowen Xu, Weikai Li, Jian Mei, Mengling Tao, Xiangbin Wang, Qianhua Zhao, Xiaoniu Liang, Wanqing Wu, Ding Ding, Peijun Wang

**Affiliations:** ^1^Department of Medical Imaging, Tongji Hospital, Tongji University School of Medicine, Shanghai, China; ^2^College of Computer Science and Technology, Nanjing University of Aeronautics and Astronautics, Nanjing, China; ^3^Physical Education College, Soochow University, Suzhou, China; ^4^Institute of Neurology, Huashan Hospital, Fudan University, Shanghai, China; ^5^National Clinical Research Center for Aging and Medicine, Huashan Hospital, Fudan University, Shanghai, China

**Keywords:** resting-state functional magnetic resonance imaging, functional connectivity, graph theory, multiple kernel learning, mild cognitive impairment

## Abstract

Mild cognitive impairment (MCI) is often considered a critical time window for predicting early conversion to Alzheimer’s disease (AD). Brain functional connectome data (i.e., functional connections, global and nodal graph metrics) based on resting-state functional magnetic resonance imaging (rs-fMRI) provides numerous information about brain networks and has been used to discriminate normal controls (NCs) from subjects with MCI. In this paper, Student’s *t*-tests and group-least absolute shrinkage and selection operator (group-LASSO) were used to extract functional connections with significant differences and the most discriminative network nodes, respectively. Based on group-LASSO, the middle temporal, inferior temporal, lingual, posterior cingulate, and middle frontal gyri were the most predominant brain regions for nodal observation in MCI patients. Nodal graph metrics (within-module degree, participation coefficient, and degree centrality) showed the maximum discriminative ability. To effectively combine the multipattern information, we employed the multiple kernel learning support vector machine (MKL-SVM). Combined with functional connectome information, the MKL-SVM achieved a good classification performance (area under the receiving operating characteristic curve = 0.9728). Additionally, the altered brain connectome pattern revealed that functional connectivity was generally decreased in the whole-brain network, whereas graph theory topological attributes of some special nodes in the brain network were increased in MCI patients. Our findings demonstrate that optimal feature selection and combination of all connectome features (i.e., functional connections, global and nodal graph metrics) can achieve good performance in discriminating NCs from MCI subjects. Thus, the combination of functional connections and global and nodal graph metrics of brain networks can predict the occurrence of MCI and contribute to the early clinical diagnosis of AD.

## Introduction

Alzheimer’s disease (AD) is a progressive neurodegenerative disorder characterized by loss of memory and cognitive decline (Blennow et al., 2006). With the aging of the global population, there will be an estimated 115 million AD patients in the world by 2050, with an average of 1 new AD patient every 33 s (Ijaopo, 2017). Mild cognitive impairment (MCI) is an intermediate stage that precedes early AD. Evidence indicates that about 15% of MCI patients progress to AD per year (Petersen et al., 1999; Grundman et al., 2004). Therefore, MCI is regarded as the critical time window for early prediction of conversion to AD (Manly et al., 2008).

Components of the brain functional connectome, including functional connections and graph theory topological metrics, have become important imaging markers for exploring brain networks and predicting the classification of neurodegenerative diseases (Biswal et al., 2010; Wang et al., 2013; Filippi et al., 2018). The functional connectome systematically depicts global graph metrics (i.e., small world, modularity, global efficiency), nodal graph metrics (i.e., degree, participant coefficient, shortest path length), and functional connections of the network. It provides a novel approach for revealing altered brain network patterns (delEtoile and Adeli, 2017; Khazaee et al., 2017; Filippi et al., 2018). Given the large numbers of network features in the brain connectome, the Student’s *t*-test (Qiao et al., 2016; Li W. et al., 2019) and sparse methods such as least absolute shrinkage and selection operator (LASSO) have been applied to select the critical features of brain networks (Wee et al., 2014; Li Y. et al., 2019). Nodal graph metrics naturally have a group topology (i.e., a node corresponds to a group of node-graph theoretical attributes). Group-LASSO is a regression-analysis method for group-feature selection and regularization that can be adopted to select nodal graph metrics (Liu et al., 2019) and maintain significant discrimination of nodal features.

In recent years, machine learning approaches with data-driven algorithms have been used to combine and classify brain features. Some classifiers such as support vector machines (SVMs) (Prasad et al., 2015; Khazaee et al., 2016), Naïve Bayes (Zhuo et al., 2018) and deep neural networks (Themistocleous et al., 2018) are applied to discriminate normal controls from subjects with MCI. However, most of these methods focus on a single modality of imaging, the functional connectome, or graph theory attributes separately, resulting in relatively poor classification performance (Suk et al., 2014). Therefore, the multimodal brain network (i.e., functional connections and graph theory topological metrics) should be used to provide a comprehensive and insightful understanding of the brain network in patients with MCI. Combined with information from different attributes, multiple kernel learning SVM (MKL-SVM) (Niu et al., 2017) can partially alleviate the high-dimensional curve of multiple features and measure the contributions of different features to the classification. These proposed methods could help select critical features and discriminate normal controls from subjects with diseases.

The main purposes of the present study were to select discriminative features of the brain connectome (i.e., functional connections, global graph metrics, and nodal graph metrics) and develop a classification of MCI based on different attributes of the brain network. Altered patterns of discriminative features were further analyzed using the proposed methods. By combining the group-LASSO model and MKL-SVM, we (i) identified the most discriminative nodal features of the brain connectome and predominant brain regions in MCI patients, (ii) achieved accurate and automatic classification of MCI patients and normal controls (NCs), and (iii) analyzed the changed patterns in the brain network.

## Materials and Methods

### Participants

Participants with MCI and NCs were recruited to establish a registry at Huashan Hospital. Each participant underwent a comprehensive evaluation, including clinical interview, neuropsychological assessment, laboratory tests, and multimodal magnetic resonance imaging (MRI) examinations of the brain. MCI was defined according to the following criteria (Petersen, 2004): (i) cognitive concern/complaint by the subject, nurse, or physician, with a Clinical Dementia Rating (CDR) = 0.5; (ii) objective impairment in ≥1 cognitive domain based on 1.5 standard deviations (SDs) below the mean using the norms obtained in the pilot study; (iii) basic normal functional activities (determined by CDR and daily living activity assessment); (iv) absence of dementia according to the Diagnostic and Statistical Manual of Mental Disorders, 4th edition (Rabe-Jablonska and Bienkiewicz, 1994). The inclusion criteria of NCs were: (i) no neurology-related or cerebral vascular diseases (e.g., Parkinson’s disease, intracranial aneurysms, or cerebral tumors); (ii) no severe mental retardation or schizophrenia; (iii) no severe problems in speaking, vision, or hearing; (iv) able to actively participate in the neuropsychological assessment. In the present study, 105 participants (41 MCI patients and 64 NCs) were selected. Two patients with MCI and four NCs were excluded due to incomplete data in resting state-functional MRI (rs-fMRI) and severe head motion at some time points. Finally, data from 99 individuals (39 MCI patients and 60 NCs) were included in the subsequent statistical analyses. The clinical and demographic data of these 99 participants were summarized. The study protocol was approved by the Ethics Committee of Huashan Hospital of Fudan University (Shanghai, China). Written informed consent was obtained from each participant (or his/her legal representative). In addition, we adopted the Alzheimer’s Disease Neuroimaging Initiative (ADNI)^1^ dataset as an independent test dataset to verify the performance of the pre-trained model.

### Data Acquisition

Rs-fMRI and structural MR images were acquired on a 3T MR scanner (Magnetom^®^ Verio; Siemens, Munich, Germany) using a 32-channel head coil. Before imaging, all participants were instructed to keep their eyes closed (but not to fall asleep), think of nothing, and move as little as possible during data acquisition. Three-dimensional (3D) T1-weighted sagittal images were acquired using magnetization-prepared rapid gradient echo with the following parameters: repetition time (TR) = 2530 ms, echo time (TE) = 2.34 ms, flip angle = 7°, inversion time (TI) = 1100 ms, matrix = 256 × 256, slice number = 192, thickness = 1.0 mm, and voxel size = 1 × 1 × 1 mm^3^. The scan lasted 6 min 03 s. The parameters of the rs-fMRI protocol were as follows: axial acquisition, TR = 2000 ms, TE = 30 ms, flip angle = 90°, slice thickness = 3.8 mm, slice number = 31, field of view = 220 × 220 mm^2^, matrix size = 64 × 64, and voxel size = 3.4 × 3.4 × 3.8 mm^3^. Each scan collected 240 volumes with a scan time of 8 min 06 s. The ADNI dataset was acquired on the 3T Philips with the following scan parameters: TR = 3000 ms, TE = 30 mm, flip angle = 80°, slice thickness = 3.3 mm, slice number = 48, matrix size = 64 × 64, and measurements = 140.

### Image Preprocessing

Preprocessing procedures were carried out using Data Processing Assistant for Resting-State fMRI (DPARSF)^2^ and Statistical Parametric Mapping (SPM12)^3^. The first 10 time points were not used to ensure stabilization of the initial signal and adaptation of participants to the environment. Timing correction to the last slice was conducted. Realignment for compensation of head-movement effects was achieved using a six-parameter rigid-body spatial transformation. All spatial movement was <3 mm of displacement and <3° of rotation in any direction, and no participant was excluded. Next, rs-fMRI images were co-registered to the high-resolution 3D-T1 structural images. Normalization of 3D-T1 structural MRI images to Montreal Neurological Institute (MNI) space was undertaken by non-linear warping based on Diffeomorphic Anatomical Registration Through Exponentiated Lie Algebra (DARTEL). Then, rs-fMRI images were spatially normalized to the MNI space using the parameters derived from the normalization of structural images and simultaneously resampled into 3-mm isotropic voxels. All normalized fMRI images were smoothed with a 6-mm, full-width at half-maximum Gaussian kernel. Linear detrending and band-pass filtering at 0.01–0.1 Hz were carried out to control low-frequency drift and high-frequency physiological noise. Finally, nuisance covariates were regressed out, including the Friston 24-motion parameter model (six head-motion parameters, six head-motion parameters one time point before, and the 12 corresponding squared items), global mean, white matter, and cerebrospinal fluid signals.

### Brain Network Construction

The average time series within each region based on the 264 putative functional area atlas were separately extracted to construct the connectivity brain network (Power et al., 2011). The Pearson’s correlation coefficients of all pairs of 264 regions of interest (ROIs) were applied separately to define the edges of functional connections. Thus, the functional connectivity matrix (adjacency matrix) was constructed (Li et al., 2017). The final functional connection networks produced N^∗^(N-1)/2 edges, where N corresponded to the number of nodes in the networks. Considering the ambiguous interpretation of negative correlations, we restricted the analysis to positive correlations and set the negative correlation coefficients as zero. A thresholding method based on network sparsity was adopted to remove the less significant connections and to retain the topological properties of graph theory by setting an appropriate threshold for network sparsity (Dai et al., 2019). Sparsity thresholds (ranging from 0.02 to 0.5, with steps of 0.01) were set to acquire a binary undirected network (Chang et al., 2016). To avoid ambiguity, we used the area under the curve (AUC; i.e., the sum value of 49 values of the corresponding node attributes) as input for the node attribute to train the classifier.

### Computation of Graph Metrics

Based on binary undirected matrices, we systematically analyzed the global and local properties of the functional brain network with the Graph Theoretical Network Analysis Toolbox (GRETNA)^4^ based on Statistical Parametric Mapping (SPM8; see text footnote 3) with MATLAB R2013b. Global metrics [i.e., clustering coefficient (C_p_), characteristic path length (L_p_), normalized clustering coefficient (γ), normalized characteristic path length (λ), small-world σ, global efficiency (E_global_)], and nodal properties (i.e., degree centrality, nodal efficiency, betweenness centrality, shortest path length) were applied to characterize the different patterns of connections in the brain network (Table 1; Wang et al., 2015). The modularity (*Q*) of a brain network quantified the efficiency of segmenting a network into modules (Newman, 2006). A modified greedy optimization algorithm was used as follows:

$$Q=\sum_{i=1}^{N_{m}}\left[l_{i}/L-{\left(d_{i}/2\,L\right)}^{2}\right]$$

**TABLE 1:** Global and local graph metrics of the brain connectome.

Global graph metrics	Local graph metrics
Clustering coefficient C_p_	Betweenness centrality
Characteristic path length L_p_	Degree centrality
Normalized clustering coefficient γ	Nodal clustering coefficient
Normalized characteristic path length λ	Local efficiency
Small-world σ	Shortest path length
Network efficiency	Participant coefficient
Modularity	Within-module degree

where *N*_*m*_ represents the number of modules, *L* is the total number of edges in the brain network, and *l*_*i*_ is the number of within-module edges in module *i*; *d*_*i*_ represents the sum of the linked edges at each node within module *i*. Modified greedy optimization was applied to detect the modular structure (Newman, 2004).

At the module level, the intra-module connectivity density (*D*_*s*_) and intermodule connectivity density (*D*_*s,t*_) were calculated as follows:

$$D_{s}=\frac{2\,\sum_{i,j\in s}\varepsilon_{i,j}}{N_{s}\,\left(N_{S}-1\right)}$$

where *N*_*S*_ is the number of nodes within module s, and ε_*i,j*_ are the edges within module *s*.

$$D_{s,t}=\frac{\sum_{i\in s,j\in t}\varepsilon_{i,j}}{N_{s}*N_{t}}$$

where *N*_*s*_ is the number of nodes within module *s*, *N*_*t*_ represent the number of nodes within module *t*, and ε_*ij*_ is the number of edges between module *s* and module *t*.

Moreover, at the nodal level, within-module degree (WD) and the participation coefficient (PC) were measured as follows:

$$W\,D_{i}=\frac{e_{i}-{\bar{e}}_{s}}{\sigma_{s}}$$

where *e*_*i*_ is the nodal degree of node *i* within module s, ${\bar{e}}_{s}$ is the average nodal degree of all nodes in module s, and σ_*s*_ is the standard deviation of the nodal degree within the module of all nodes in module s.

$$P\,C_{i}=1-\sum_{s=1}^{N_{m}}{\left(\frac{k_{i,s}}{k_{i}}\right)}^{2}$$

where *N*_*m*_ is the number of modules and *k*_*i,s*_ is the number of connections between node *i* and module *s*. *k*_*i*_ represents the number of connections of node *i* to all other nodes within the *N*_*m*_ modules.

Nodes with a degree of 2 standard deviations higher than the mean of the degree of all nodes were identified as hub nodes (Rubinov and Sporns, 2010). Small-world attributes were applied to characterize an optimized balance between functional segregation and integration of the network.

### Statistical Analyses

For demographics and clinical characteristics, two-sample Student’s *t*-tests were carried out except for sex, which was tested by the chi-square test. *P* < 0.05 indicated a significant difference in the demographic data. First, functional connections and global and local metrics were regressed to remove potential effects of the covariates age, sex, and education duration. Then, differences pertaining to graph theory metrics between MCI patients and NCs were compared based on two-sample Student’s *t*-tests. A procedure to ascertain the false discovery rate was performed to further correct for multiple comparisons. To localize the specific pairs of regions in which functional connections were altered in MCI patients, we used a network-based statistic (NBS) approach (Zalesky et al., 2010). A corrected *P*-value was calculated for each component using the null distribution of the maximal connected component size, which was empirically derived using a non-parametric permutation approach (10,000 permutations) (Zuo et al., 2012). *P* < 0.01 indicated a significant difference.

### Feature Selection for Nodal Graph Metrics

As mentioned above, the brain was divided into 264 nodes based on the 264 putative functional area atlas (Power et al., 2011), and each node corresponded to seven local graph metrics (i.e., betweenness centrality, degree centrality, nodal clustering coefficient, local efficiency, shortest path length, participant coefficient, within-module degree). Thus, the nodal graph metrics naturally have a group topology, that is, a node corresponds to a group of node-graph theoretical attributes. Given the natural group attributes, we used group-LASSO as the feature-selection scheme for nodal graph metrics.

$$m\,i\,n_{W}\,\sum_{i=1}^{n\,S\,u\,b}l\,o\,g\,\left(1+e\,x\,p\,\left(-y_{i}\times \left(\sum_{j=1}^{n\,R\,O\,I}\sum_{k=1}^{7}w_{\left(j,k\right)}\,x_{\left(j,k\right)}+c\right)\right)\right)\,+\lambda \,\sum_{j=1}^{n\,R\,O\,I}{||w_{j\,k}||}_{q},$$

where *y*_*i*_ is the label of the *i*-th participant, and *w*_*(j,k)*_ and *x*_*(j,k)*_ are the weight and value of the *j*-th ROI and *k*-th Nodal Graphic Metric, respectively. Note that *x*_*(j,k)*_ is normalized by Fisher Z-transformation to avoid scale imbalance. We used the SLEP toolbox^5^ to calculate *w*_*(j,k)*_ with a default setting of λ = 1.

### Classification

Combination of information provides an effective way to integrate multiple views of biomarkers (i.e., connections and graph metrics). The simplest way is to overlay the data directly, but this approach can be inappropriate due to the high-dimensional curve and small number of samples. Moreover, a modality with more dimensions can submerge a modality with fewer dimensions. To overcome this challenge, we used MKL-SVM for information combination because the kernel trick can partially alleviate the high-dimensional curve. MKL-SVM was conducted as shown below.

Suppose that there are n training samples with connection values and graph metrics. For $x_{i}^{m}$, *m* = 1,2,3, which correspond to the connection value, the nodal graph metrics and global graph metrics respectively. *y* represent the correcponding class label of the *i*-th sample. MKL-SVM solves the following primal problem:

$$\min_{w}\frac{1}{2}\,\sum_{m=1}^{2}\beta_{m}\,{||w^{m}||}^{2}+C\,\sum_{i=1}^{2}\xi_{i}$$

$$s.t.y_{i}\,\left(\sum_{m=1}^{2}\beta_{m}\,{\left(w^{m}\right)}^{T}\,\phi^{m}\,\left(x_{i}^{m}\right)+b\right)\geq 1-\xi_{i}$$

$$\xi_{i}\geq 0,i=1,2$$

where ϕ^*m*^ represents a mapping from the original space to the Represent Hilbert Kernel Space (RHKS), *w^m^* represents the normal vector of the hyperplane in RHKS, and β_*m*_ denotes the corresponding combining weight on the m-th modality. Then, the dual form of MKL-SVM can be represented as:

$$\max_{\alpha }\,\sum_{i=1}^{n}\alpha_{i}-\frac{1}{2}\,\sum_{i,j}\alpha_{i}\,\alpha_{j}\,y_{i}\,y_{j}\,\sum_{m=1}^{2}\beta_{m}\,k^{m}\,\left(x_{i}^{m},y_{i}^{m}\right)$$

$$s.t.\sum_{\text{i}=1}^{\text{n}}\alpha_{i}\,y_{i}=0$$

$$0\leq \alpha_{\text{i}}\leq C,i=1,2$$

where $k^{m}\,\left(x_{i}^{m},y_{i}^{m}\right)=\phi^{m}\,{\left(x_{i}^{m}\right)}^{T}\,\phi^{m}\,\left(x_{j}^{m}\right)$ and is the kernel matrix on the m-th modality. After we trained the model, we tested the new samples *x* = {*x*_1_, *x*_2_, …, *x*_*M*_}. The kernel between the new test sample and the *i*-th training sample on the m-th modality is defined as $k^{m}\,\left(x_{i}^{m},x^{m}\right)=\phi^{m}\,{\left(x_{i}^{m}\right)}^{T}\,\phi^{m}\,\left(x^{m}\right)$. In the end, the predictive level based on MKL-SVM can be formulated as follows:

$$f\,\left(x_{1},x_{2},\ldots ,x_{M}\right)=\text{sign}\,\left(\sum_{\text{i}=1}^{\text{n}}y_{i}\,\alpha_{i}\,\sum_{m=1}^{M}\beta_{m}\,k^{m}\,\left(x_{i}^{m},x^{m}\right)+b\right)$$

**FIGURE 1:**
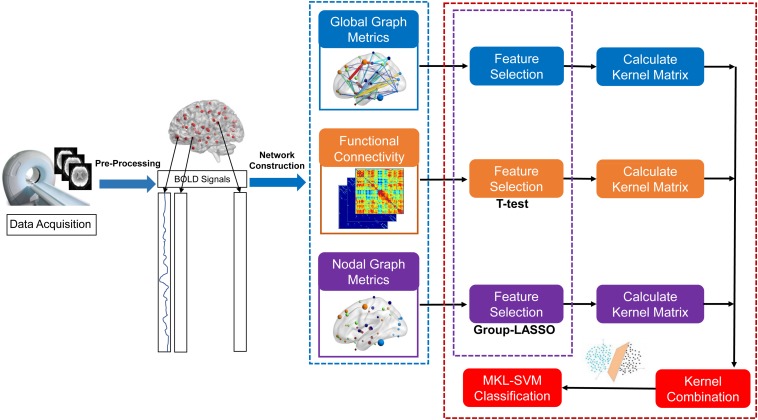
Data-processing and classification procedures employed in our study.

The proposed formulation of MKL-SVM is similar to but different from existing multi-kernel learning methods because β_*m*_ is selected based on the cross-validation scheme on the grid-searching space with constraints ∑_m_ β_*m*_ = 1. The range of c was 2^∧^−5 to 2^∧^5. All data-processing and classification procedures used in our study are shown in Figure 1. Due to the small sample size, we used the leave-one-out cross-validation (LOOCV) strategy to verify the performance of the methods, in which only one subject is left out for testing while the others are used to train the models and obtain the optimal parameters. For the choice of optimal parameters, an inner LOOCV was conducted on the training data using a grid-search strategy. Moreover, in order to verify the performance of the proposed model, we also tested the model on the independent ADNI dataset.

## Results

### Demographics and Clinical Characteristics

The demographic data and clinical characteristics of all participants are summarized in Table 2. There were no significant differences in sex, age, or education level between the MCI and NC groups (*P* > 0.05 for all). However, the MCI group had significantly lower scores on the Mini Mental State Examination (*P* < 0.001) than the NC group. We also selected 50 samples (27 MCI and 23 NCs) from the independent ADNI dataset. The details of their demographic and clinical characteristics are listed in Table 3.

**TABLE 2:** Demographics and clinical characteristics of MCI patients and NCs in the current study.

Characteristic	MCI (*n* = 39)	NCs (*n* = 60)	*P*
Male/female, *n*	25/14	30/30	0.168^a^
Age, years, mean ±SD	74.00 ± 7.67	71.25 ± 7.08	0.071^b^
Education, years, mean ±SD	10.97 ± 4.29	12.42 ± 3.58	0.074^b^
MMSE, mean ±D	26.77 ± 2.33	28.28 ± 1.35	<0.001^b^
Hippocampal volume (×10^3^ mm^3^)	6.80 ± 0.87	7.43 ± 0.69	0.002^b^

*MMSE, Mini mental state examination. ^*a*^The *P*-value was obtained by using the chi-square test. ^*b*^The *P*-value was obtained by using a two-sample *t*-test.*

**TABLE 3:** Demographics and clinical characteristics of the ADNI dataset.

Characteristic	MCI (*n* = 27)	NCs (*n* = 23)	*P*
Male/female, *n*	13/14	11/12	0.982^a^
Age, years, mean ±SD	70.11 ± 8.17	75.22 ± 6.82	0.021^b^
MMSE, mean ±SD	25.33 ± 1.07	27.17 ± 1.30	<0.001^b^

*MMSE, Mini mental state examination. ^*a*^The *P*-value was obtained by using the chi-square test. ^*b*^The *P*-value was obtained by using a two-sample *t*-test.*

### Significant Differences of Functional Connections in Brain-Network

**FIGURE 2:**
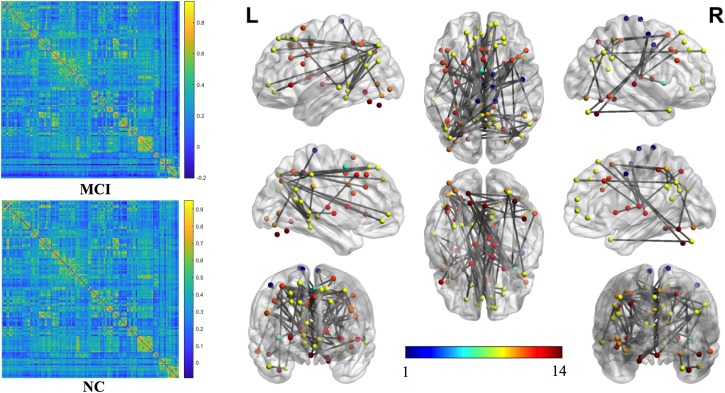
The most significant 100 connections mapped on the ICBM 152 template using the BrainNet Viewer software package (http://nitrc.org/projects/bnv/). The connectivity matrices of the fully connected network of MCI patients and NCs are shown. The 100 most significant connections were retained, with gray indicating a reduction in connectivity strength. Plots in this figure were created by BrainNet Viewer (http://nitrc.org/projects/bnv/). The color-bar numbers represent the subnetworks with reference to the 264 putative functional area atlas proposed by Power et al. (2011). The details are: 1 sensory/somatomotor hand network; 2 sensory/somatomotor mouth network; 3 cingulo-opercular task control network; 4 auditory network; 5 default mode network; 6 memory retrieval network; 7 visual network; 8 frontoparietal task control network; 9 salience network; 10 subcortical network; 11 ventral attention network; 12 dorsal attention network; 13 cerebellar network; 14 unknown network.

The mean connection strengths of the whole brain network were compared between MCI and NC. A total of 3072 connections with significant differences were extracted between the MCI and NC groups within the range of fully sparse values from 0.02 to 0.5 (*P* < 0.01) using Student’s *t*-tests. After permutation of NBS, we retained the most significant 100 connections with the lowest *P*-values (Figure 2). We projected them into the corresponding subnetworks and found that the most discriminative network connections were mainly distributed in the default mode network (DMN), subcortical network, frontoparietal task control network, dorsal attention network, and visual network. Compared with NCs, patients with MCI had significantly lower functional connection strength in brain-network connections (*P* < 0.01).

### Global Graph Metrics of the Functional Brain Connectome

**FIGURE 3:**
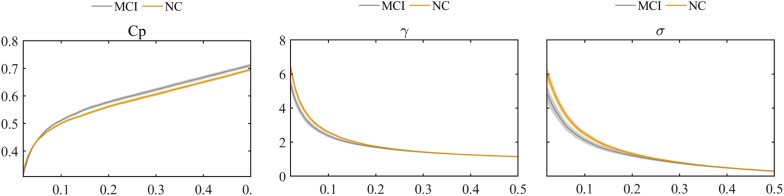
Comparison of clustering coefficient (C_p_), normalized clustering coefficient (γ), and small-world σ between MCI and NC groups.

The global graph metrics of the MCI and NC groups showed the small-world topological attributes. That is, the functional brain networks had larger clustering coefficients and almost identical shortest path lengths compared with the matched random networks. With increasing connection density, C_p_ increased, whereas L_p_, γ, λ, and small-world σ decreased in the MCI and NC groups. Statistical analysis revealed that the C_p_ of MCI patients was higher than that in the NC group, whereas λ and small-world σ were lower in the MCI group compared with the NC group (*P* < 0.01). However, these differences were only observed at a few network thresholds (Figure 3).

### Nodal Graph Metrics of the Functional Brain Connectome

**FIGURE 4:**
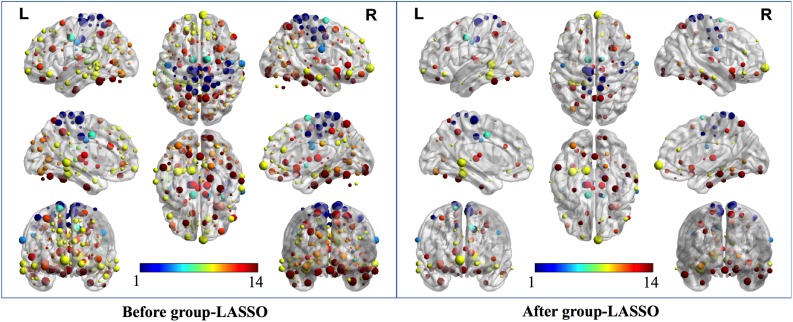
The most predominant nodes for discriminating MCI patients from NCs. Before group-LASSO, 212 significantly different nodes were present between MCI and NC groups (*P* < 0.01). After feature selection by group-LASSO, the 76 most highly discriminative nodes were reserved. The color-bar numbers represent the subnetworks with reference to the 264 putative functional area atlas proposed by Power et al. (2011). The details are: 1 sensory/somatomotor hand network; 2 sensory/somatomotor mouth network; 3 cingulo-opercular task control network; 4 auditory network; 5 default mode network; 6 memory retrieval network; 7 visual network; 8 frontoparietal task control network; 9 salience network; 10 subcortical network; 11 ventral attention network; 12 dorsal attention network; 13 cerebellar network; 14 unknown network.

Two strategies were developed to investigate the discriminative features of nodal graph metrics and nodes based on local network parameters. On the one hand, we analyzed the most predominant brain regions with the greatest number of significant differences in nodal graph metrics. Before group-LASSO, 212 significantly different nodes were observed between MCI and NC groups (*P* < 0.01). However, after feature selection by group-LASSO, we selected the nodal graph metrics from 76 ROIs as inputs. These 76 ROIs were considered as the extremely predominant nodes for discriminating MCI patients from NCs, and each ROI had ≥4 and ≤7 nodal topological metrics with significant differences. The locations of nodes in the 264 atlas were labeled according to the AAL_90 atlas (Figure 4 and Table 4). On the other hand, we identified the distinguishing features for each nodal graph theory attribute using the feature selection of group-LASSO (Table 5). The top-20 nodal graph topological features with maximum discriminative ability are listed in Table 6. Therefore, the most predominant brain regions with the greatest numbers of significant nodal graph measures and the most discriminative nodal graph features were distributed mainly in the temporal, cingulate, superior frontal, lingual, and parietal gyri, which corresponded to the DMN, dorsal attention network, and cingulo-opercular task network.

**TABLE 4:** Top 20 most predominant nodes (brain regions) with the greatest number of significant differences in nodal graph metrics.

ROI number	Corresponding AAL area	Sub-network	Number of nodal metrics
77	Lingual_L	Default mode	7
126	Fusiform_L	Default mode	7
4	Temporal_Inf_L	Unknown	7
116	Temporal_Mid_R	Default mode	7
22	Precuneus_R	Sensory/somatomotor	6
17	Paracentral_Lobule_L	Sensory/somatomotor	6
251	Precuneus_R	Dorsal attention	6
259	Parietal_Inf_L	Dorsal attention	6
75	Frontal_Mid_Orb_R	Default mode	6
92	Cingulum_Post_R	Default mode	6
224	Thalamus_L	Subcortical	6
225	Thalamus_R	Subcortical	6
53	Supp_Motor_Area_R	Cingulo-opercular task	6
211	Insula_R	Salience	6
203	Cingulum_Mid_R	Salience	6
124	ParaHippocampal_L	Default mode	6
139	Frontal_Inf_Orb_R	Default mode	5
51	Cingulum_Mid_L	Cingulo-opercular task	5
172	Fusiform_L	Visual	5
263	Parietal_Sup_L	Dorsal attention	5

*AAL, the automated anatomical labeling atlas.*

**TABLE 5:** Number of discriminative features for each nodal graph metrics from the feature-selection step of LASSO.

Nodal graph metric	Number of selected features
Betweenness centrality	33
Degree centrality	46
Nodal clustering coefficient	48
Nodal local efficiency	19
Nodal shortest path length	44
Participant coefficient	70
Within-module degree	81

**TABLE 6:** Top 20 features corresponding to nodal graph metrics with maximum discriminative ability.

Nodal graph measure	ROI number	Corresponding AAL area	Subnetwork
Within-module degree	124	ParaHippocampal_L	Default mode
Within-module degree	89	Precuneus_R	Default mode
Within-module degree	191	Parietal_Sup_L	Frontoparietal task
Degree centrality	77	Lingual_L	Default mode
Participant coefficient	9	Temporal_Inf_R	Uncertain
Within-module degree	92	Cingulum_Post_R	Default mode
Degree centrality	225	Thalamus_R	Subcortical
Participant coefficient	118	Temporal_Mid_L	Default mode
Participant coefficient	75	Frontal_Mid_Orb_R	Default mode
Nodal clustering coefficient	75	Frontal_Mid_Orb_R	Default mode
Nodal shortest path length	75	Frontal_Mid_Orb_R	Default mode
Participant coefficient	17	Paracentral_Lobule_L	Sensory/somatomotor
Degree centrality	224	Thalamus_L	Subcortical
Nodal shortest path length	9	Temporal_Inf_R	Uncertain
Participant coefficient	83	Temporal_Inf_L	Default mode
Degree centrality	126	Fusiform_L	Default mode
Betweenness centrality	77	Lingual_L	Default mode
Nodal clustering coefficient	77	Lingual_L	Default mode
Betweenness centrality	51	Cingulum_Mid_L	Cingulo-opercular task
Nodal local efficiency	92	Cingulum_Post_R	Default mode

*AAL, the automated anatomical labeling atlas.*

**FIGURE 5:**
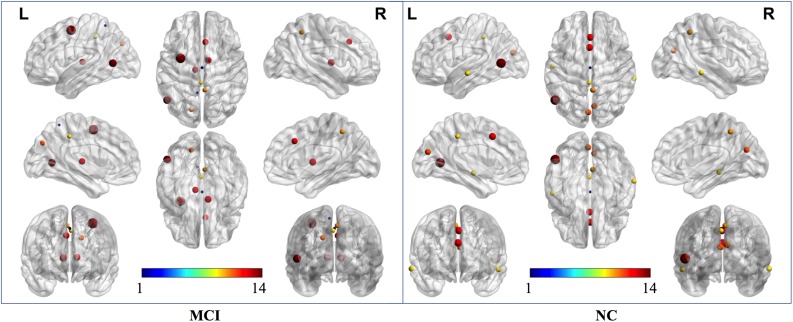
Hub nodes of MCI and NC groups in the brain. The color-bar numbers represent the subnetworks with reference to the 264 putative functional area atlas proposed by Power et al. (2011). The details are: 1 sensory/somatomotor hand network; 2 sensory/somatomotor mouth network; 3 cingulo-opercular task control network; 4 auditory network; 5 default mode network; 6 memory retrieval network; 7 visual network; 8 frontoparietal task control network; 9 salience network; 10 subcortical network; 11 ventral attention network; 12 dorsal attention network; 13 cerebellar network; 14 unknown network.

According to the definition of “hubs,” we identified hub nodes in MCI patients and NCs. Figure 5 shows the hub nodes in each group. In MCI patients and NCs, the common hub regions were mainly located in the left middle temporal gyrus, right precuneus, left median cingulate gyrus, left cuneus, and paracingulate gyri. More importantly, some hub nodes were present only in MCI patients and absent in NCs: the left paracentral lobule, right paracentral lobule, left postcentral gyrus, and right cuneus. Simultaneously, there were also some hub nodes in NCs but not in MCI patients. These regions were located on the left Heschl, right superior temporal, left inferior occipital, and left middle occipital gyri. Hub nodes play critical roles in maintaining high-level cognitive functions by coordinating overall information flow and supporting the integrity of the brain connectome (Wang et al., 2013). The similar distributions suggested preservation of hubs in MCI.

**FIGURE 6:**
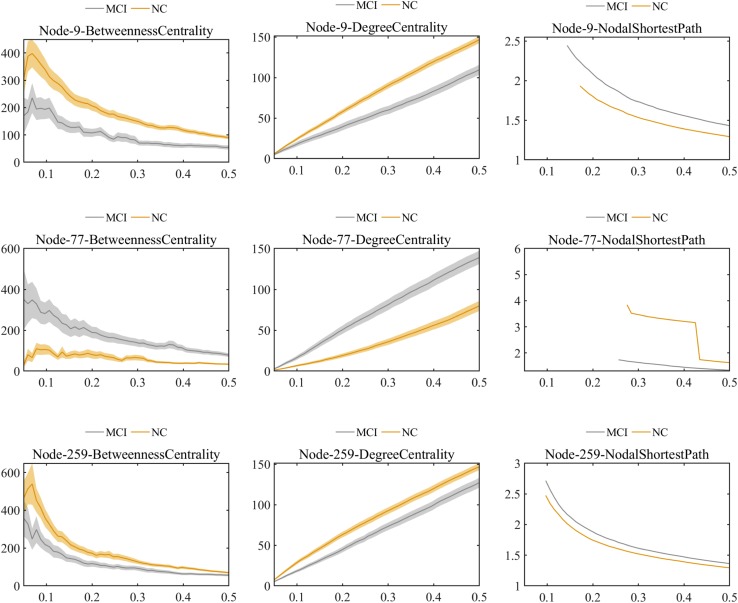
Comparison of values of nodal graph metrics between MCI patients and NCs. Betweenness centrality, degree centrality, and nodal shortest path length of Node 9 (right inferior temporal gyrus). Betweenness centrality, degree centrality and nodal shortest path length of Node 259 (left inferior parietal). Betweenness centrality, degree centrality, and nodal shortest path length of Node 77 (left lingual gyrus).

Further comparisons of the predominant brain regions mentioned above revealed that MCI patients had significantly lower values of betweenness centrality and degree centrality and significantly higher values for the nodal shortest path in the frontal lobe (e.g., bilateral superior frontal gyrus), temporal lobe (e.g., bilateral inferior temporal gyrus), limbic lobe (e.g., left median cingulate and paracingulate gyri), and parietal lobe (e.g., left inferior parietal gyrus) compared with the NC group (*P* < 0.01 for all). Nevertheless, in the occipital lobe (e.g., left lingual and left fusiform gyri), the MCI group showed significantly higher values of betweenness centrality and degree centrality and significantly lower values of nodal shortest path, which was opposite to the pattern of nodal graph metrics in the brain lobes mentioned above (Figure 6).

### Classification

**FIGURE 7:**
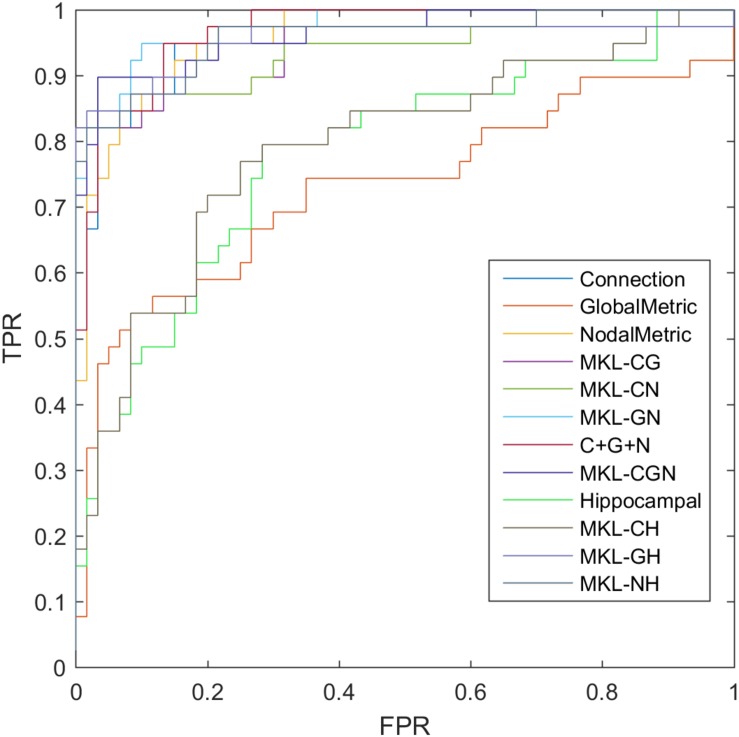
ROC of classification based on different features. C, connection; G, global metrics; N, nodal metrics; H, hippocampal volume; MKL, multiple kernel learning; FPR, false positive rate; TPR, true positive rate.

After feature selection of functional connections with Student’s *t*-tests and nodal graph metrics by group-LASSO, MKL-SVM was carried out to combine the brain connectome information. We evaluated the classification performance of different methods with a set of quantitative measures – accuracy, sensitivity, and specificity – which were defined as follows:

$$A\,c\,c\,u\,r\,a\,c\,y=\frac{T\,P+T\,N}{T\,P+F\,P+T\,N+F\,N},$$

$$S\,e\,n\,s\,i\,t\,i\,v\,i\,t\,y=\frac{T\,P}{T\,P+F\,N},$$

$$S\,p\,e\,c\,i\,f\,i\,c\,i\,t\,y=\frac{T\,N}{T\,N+F\,P},$$

where TP, TN, FP, and FN denote the number of true-positive, true-negative, false-positive, and false-negative values, respectively. The area under the receiver operating characteristic curve (AUC) was calculated as a performance measure for binary classification of the MCI and NC groups. In particular, LOOCV was employed in this study due to the small sample size, which provided an optimistic estimate of the classification accuracy since all except one of the subjects are used to train the classifier. For other approaches such as k-fold cross-validation, only N-k (N is the total number of participants in the dataset) participants are included during the training process, resulting in poorer performance due to the small dataset (Wee et al., 2012). For the functional connections (C), global metrics (G), and nodal metrics (N) of the brain network, we obtained AUCs of 0.9605, 0.7290, and 0.9576, respectively (Table 7). We also performed classification experiments by combining functional connections (C), global metrics (G), nodal metrics (N), global metrics (G), and nodal metrics (N). The results showed that despite the low classification performance of single global graph metrics, they still effectively increased the classification performance of nodal graph metrics and functional connections. For a direct combination of connections, global metrics, and nodal metrics, we obtained 87.88% accuracy and an AUC of 0.9666, which meant that simple combination did not effectively improve the classification performance. Finally, the combination of all connectome features based on MKL-SVM achieved the best classification performance, with 92.93% accuracy, 95.00% specificity, and an AUC of 0.9728. Moreover, the weight values (β) of functional connections, global metrics, and nodal metrics were 0.3, 0.01, and 0.6, respectively, indicating that the node attributes contributed most to the classification (Figure 7). It should be noted that MKL-SVM both combines the information of functional connectivity and graph theory attributes and provides a method to merge more useful information for MCI identification. Therefore, we also combined the traditional unimodal marker of hippocampal volume with the brain connectome; the results are listed in Table 7. Our results suggest that the AUC of the hippocampal volume was 0.7005, and the AUCs of the combination of hippocampal volume with functional connectivity, global graph theory attributes, or node graph theory attributes were 0.9509, 0.8117, and 0.9647, respectively. In addition, the independent ADNI dataset was then employed to verify the generalization of the pre-trained model. The all connectome features combination based on MKL-SVM achieved classification performance with 66.00% accuracy, 70.37% sensitivity, and 60.87% specificity.

**TABLE 7:** The evaluation of classification performance corresponding to different functional connectome features.

Method	Accuracy (%)	Sensitivity (%)	Specificity (%)	AUC
Connection (C)	85.86	82.05	88.33	0.9605
Global Metrics (G)	73.74	69.23	76.67	0.7290
Nodal Metrics (N)	87.88	82.05	91.67	0.9576
MKL_CG	86.87	82.05	90.00	0.9329
MKL_CN	90.91	84.62	95.00	0.9581
MKL_GN	89.90	84.62	93.33	0.9371
C + G + N	87.88	92.31	85.00	0.9666
MKL_CGN	92.93	89.74	95.00	0.9728
Hippocampal (H)	72.73	71.67	74.36	0.7005
MKL_CH	86.86	84.62	88.33	0.9509
MKL_GH	76.77	73.33	82.05	0.8117
MKL_NH	89.90	87.18	91.67	0.9647

*MKL-SVM, multiple kernel learning support vector machine.*

## Discussion

In the present study, we selected discriminative features from different attributes of the brain connectome (i.e., functional connections, global graph metrics, and nodal graph metrics) and combined the information to train a classifier for distinguishing subjects with MCI from NCs. Based on the feature selection and combination of the proposed methods, we further described the altered patterns of the best distinguishing features of MCI through group comparison, aiming to further clarify disease pathogenesis. Our detailed results are listed as follows. First, the most predominant brain regions and most discriminative nodal graph metrics for discriminating NCs from MCI were selected by the group-LASSO. Second, the information combination strategy (MKL-SVM) effectively improved the classification performance, and the nodal graph metrics of the connectome contributed most to the classification. Finally, the altered functional brain connectome pattern in MCI patients included a general decrease in functional connections in the whole brain network, whereas nodal topological attributes in some local brain regions were increased.

### The Most Predominant Brain Regions and Discriminative Nodal Graph Metrics

The nodal graph metrics have a natural group topology; that is, a node corresponds to a group of node-graph theoretical attributes. Thus, we used group-LASSO as the feature-selection scheme for nodal graph metrics. It effectively extracted the group-structure information of nodal attributes. The most predominant brain regions (with seven significantly different nodal topological metrics) were mainly distributed in the left lingual, left fusiform, left inferior temporal, and right middle temporal gyri. These brain regions showed significant changes in nodal graph metrics and so could be regarded as the most sensitive observation areas for nodal topological attributes in MCI patients. Also, within-module degree, degree centrality, and participation coefficient showed the most significant discriminative ability among the selected nodal graph metrics. The corresponding brain regions with the three most discriminative nodal metrics considerably overlapped with the hub nodes found in MCI patients. Overall, our results emphasize the importance of analyzing the attributes of intra-modules and hub nodes for early discrimination of NCs from subjects with MCI.

By projecting brain regions with significant differences of functional connections and graph metrics in the brain network to subnetworks, we found that the differences between MCI patients and NCs were distributed mainly in the DMN, dorsal attention network, cingulo-opercular task network, and frontoparietal task network. Of these, the DMN had the most significant discriminative ability. Studies have verified the correlations between these subnetworks and cognitive functions in the human brain, corresponding to spatial attention (Rolle et al., 2017), visual attention (Wirth et al., 2017), and executive function (Talpos and Shoaib, 2015).

In this study, the DMN carried the most distinguishing information, which was verified by the proposed feature selection methods. Previous studies showed that the DMN is involved in episodic memory and is considered the major cognitive domain impaired in the early stage of AD (Meskaldji et al., 2016; Dillen et al., 2017). Besides validating the discriminative ability of the DMN for discriminating NCs from MCI, we accurately located the predominant brain regions (middle temporal, inferior temporal, lingual, posterior cingulate, and middle frontal gyri) in the DMN and the corresponding nodal graph metrics. These results may facilitate the early and accurate diagnosis of MCI. They also demonstrate the repeatability and verifiability of the proposed methods, which is an important contribution of our work.

### Fusion Classification of MKL-SVM and Identification of Maximum Contribution

Group-LASSO is valid for nodal feature selection because it can retain significant features with the most discriminative ability while avoiding data redundancy. We carried out reduction of nodal features according to group-LASSO and selected optimal features to achieve the best performance for discriminating NCs from MCI. This is an effective way to integrate multiple views of biomarkers for AD classification. The simplest way is to directly splice the data. Studies using multivariate pattern analysis [e.g., linear discriminate analysis (Alam et al., 2017), artificial neural networks (Quintana et al., 2012), and random forest (Sarica et al., 2017)] have been undertaken to identify MCI using complex network characteristics. However, those approaches could be inappropriate due to the high-dimensional curves and small samples. Information with higher dimensions can submerge the low-dimension information. To overcome these challenges, we employed MKL-SVM for information combination. MKL (Niu et al., 2017) is a sparse machine-learning method that allows identification of the most relevant classification sources. The results suggested that the performance of classification by combining multiple brain connectome features was better than that of individual connectome features. The weight value (β) of functional connections, global metrics, and nodal metrics emphasized that nodal graph attributes had the greatest contribution to classification. It also indicated that MCI patients had significant changes in nodal properties. More surprisingly, although global metrics showed the worst classification performance, they can still provide important information about functional connections and nodal metrics. After combining functional connections and global metrics (C + G), functional connections and nodal metrics (C + N), and global metrics and nodal metrics (G + N), the results indicated that classification performance was effectively improved by combining the information of global metrics.

To verify this significant improvement, the Delong test was applied (DeLong et al., 1988). We found that the proposed method significantly outperformed the global graph attributes, functional connection, and nodal graph attributes under the 95% confidence interval with *P*-values of 0.0002, 0.0227, and 0.0419, respectively. Although MKL-SVM did not yield significant improvements compared to the feature concatenation method (*P* = 0.1627), it still had two advantages. First, MKL-SVM could address the imbalanced dimension issue across modalities to some extent and better embody the contribution of different information sources to distinguish MCI patients from NCs. Second, experimental results demonstrated that the proposed method outperformed the single modality of the functional connectome in the brain network. It should also be noted that both methods are simple attempts to verify information effectiveness.

The classification results based on the traditional marker of hippocampal volume suggested that the combination of hippocampal volume and connectome features could also improve classification accuracy. The MKL-SVM can be used to combine multiple features of the brain connectome and effectively integrate multimodal information to discriminate NCs from patients with MCI.

During validation of the proposed model, the classification performance of the independent ADNI dataset was not as good as the pre-trained sample. This may be due to heterogeneity in scanning machines, parameters, and physiological structures between western and eastern samples, which obviously violates the independently identically distribution assumption of SVM.

### Altered Pattern of the Brain Network Connectome in MCI

At the global brain level, we found that MCI patients had weaker functional connections in the brain network, which was consistent with previous functional network studies of AD (Li et al., 2016) and MCI (Wang et al., 2013; Lee et al., 2016). Some results demonstrated that these abnormal functional connections were directly related to the global topological attributes of brain networks (Wang et al., 2013). In our study, we first found that patients with MCI and NCs fit the features of a small-world network in a global network topology. That is, the brain network supported rapid, real-time integration of information across separate sensory brain regions to confer resilience against pathology and maximize efficiency with minimal cost for effective information processing between brain regions (Sporns and Zwi, 2004; Achard and Bullmore, 2007; Sporns, 2011). Further comparison suggested that the value of small-world σ in MCI patients was lower than that in NCs, indicating “economic small-world” disruption (Liao et al., 2017) (i.e., reduction of the segregation and integration functions of effective information in the brain network). Moreover, we found changes in the functional segregation of brain networks in MCI patients (increased C_p_). C_p_ is a measure of local network connectivity (Bullmore and Sporns, 2009) that reflects the efficiency of local information transfer and the ability to defend against random attacks against a network. A higher value of C_p_ represents a more concentrated clustering of local connections and a stronger capacity for processing local information. It is notable that previous studies reported decreased C_p_ in AD patients (Zhao et al., 2012). The reason for this difference might be related to the compensatory change of segregation function in the transition stage of MCI. Therefore, our results suggested that functional connections in the whole-brain network were generally decreased, whereas the network segregation of local information processing was increased.

At the local brain level, further analyses of the hub nodes and nodes with the most discriminative ability for MCI showed that MCI patients had significantly lower values of betweenness centrality and degree centrality and higher values of nodal shortest path in some brain regions (the frontal, temporal, limbic, and parietal lobes) compared with NCs. These data suggested that the network integration and local transmission capability of these lobes were decreased in MCI patients. However, in critical nodes in the occipital lobe, the increased betweenness/degree centrality and decreased shortest path indicated enhanced integration function and greater local transmission efficiency. We speculated that enhanced variation of these nodal graph metrics in some occipital nodes suggests compensation to maintain high-level cognitive performance despite the pathological process of amyloid accumulation during the earliest phases of AD. This functional variation in the occipital lobe was also mentioned in previous studies. For example, Dai et al. found that the left fusiform gyrus exhibited higher functional connections in the AD group (Dai et al., 2015). Bokde et al. (2010) found significantly greater activation in the right middle occipital gyrus during the location-matching task.

Therefore, the altered brain connectome patterns in our study revealed that functional connections generally decreased in the whole brain network but increased for nodal graph topological attributes of local brain regions. This might suggest functional compensation in some brain regions to maintain normal cognitive function in the early stage of AD.

### Limitations and Future Directions

There are still several limitations that need to be considered further. First, the class imbalance issue. Although there are several approaches (e.g., resampling or reweighting) to overcome imbalance, taking them makes it difficult to estimate whether the improvement of performance is based on these adjustments or on the proposed methods. In the future, we plan to investigate high-quality data with more balanced samples for feature selection and classification or develop a more robust algorithm that improves classification accuracy and generalization.

Second, we assessed a small sample size. The optimization of parameters and hyperparameters inevitably leads to overfitting for small samples. To avoid this issue, we empirically chose parameters with a default setting of lambda = 1 and *C* = 1 instead of optimized parameters and hyperparameters. In the future, we will conduct parameter optimization based on a larger sample size.

Third, we must consider the generalization of the model. For the independent ADNI dataset, classification performance was not as good as observed for the pre-trained sample, which suggests a limitation in modal generalization for different centers. We intend to improve the classification performance of multicenter data sources by combining domain adaptation.

Finally, our cross-validation approach may have been insufficient. Evaluation of classification by *k*-fold cross-validation might be more precise when sufficient data are available. Therefore, in the future, it is necessary to compare the results obtained by different cross-validation methods (i.e., LOOCV and *k*-fold cross-validation).

## Conclusion

In the present study, the discriminative features of functional connections and nodal graph metrics were selected by Student’s *t*-tests and group-LASSO, respectively. The combination of all connectome information using MKL-SVM achieved the best classification performance (AUC = 0.9728). In addition, the altered brain connectome pattern revealed that functional connectivity was generally decreased in the whole-brain network, whereas graph theory topological attributes of some special nodes were increased in MCI patients. Our findings demonstrate that optimal feature selection and the combination of all connectome features could achieve good performance for discriminating NCs from MCI. The combination of functional connections and global and nodal graph metrics of brain networks can predict the occurrence of MCI and contribute to the early clinical diagnosis of AD.

## Data Availability Statement

All datasets generated for this study are included in the article/supplementary material.

## Ethics Statement

The studies involving human participants were reviewed and approved by the Ethics Committee of Huashan Hospital within Fudan University (Shanghai, China). The patients/participants provided their written informed consent to participate in this study.

## Author Contributions

XX and PW designed the study. QZ diagnosed patients. XL and WW administered the neuropsychological tests. XW acquired the MRI data. XX, WL, and JM analyzed and interpreted the results of the data. XX and MT drafted the manuscript. DD and PW revised the manuscript. All authors contributed equally to this work and approved the final manuscript.

## Conflict of Interest

The authors declare that the research was conducted in the absence of any commercial or financial relationships that could be construed as a potential conflict of interest.

## Footnotes

1 http://adni.loni.usc.edu/

2 http://restfmri.net/forum/index.php

3 http://www.fil.ion.ucl.ac.uk/spm

4 www.nitrc.org/projects/gretna/

5 www.yelab.net/software/SLEP
